# Inequities and their determinants in coverage of maternal health services in Burkina Faso

**DOI:** 10.1186/s12939-018-0770-8

**Published:** 2018-05-11

**Authors:** Takondwa Mwase, Stephan Brenner, Jacob Mazalale, Julia Lohmann, Saidou Hamadou, Serge M. A. Somda, Valery Ridde, Manuela De Allegri

**Affiliations:** 1 Institute of Public Health, Faculty of Medicine, Heidelberg University, INF 130.3, Heidelberg, Germany; 2 University of Malawi, Chancellor College, PO Box 280, Zomba, Malawi; 3 University of Montreal Public Health Research Institute (IRSPUM), 7101 Avenue de Parc, Room 3060, Montreal, QC H3N 1X9 Canada; 4 Centre MURAZ 2054 Avenue Mamadou KONATE, 01 B.P. 390, Bobo-Dioulasso 01, Burkina Faso; 5 University of Montreal Hospital Research Centre (CRHHUM), 850 Saint Denis, 3rd Floor, Montreal, QC H2X 0A9 Canada

**Keywords:** Maternal health, Inequities, Coverage, Household wealth, Distance, Burkina Faso, Sub-Saharan Africa

## Abstract

**Background:**

Poor and marginalized segments of society often display the worst health status due to limited access to health enhancing interventions. It follows that in order to enhance the health status of entire populations, inequities in access to health care services need to be addressed as an inherent element of any effort targeting Universal Health Coverage. In line with this observation and the need to generate evidence on the equity status quo in sub-Saharan Africa, we assessed the magnitude of the inequities and their determinants in coverage of maternal health services in Burkina Faso.

**Methods:**

We assessed coverage for three basic maternal care services (at least four antenatal care visits, facility-based delivery, and at least one postnatal care visit) using data from a cross-sectional household survey including a total of 6655 mostly rural, poor women who had completed a pregnancy in the 24 months prior to the survey date. We assessed equity along the dimensions of household wealth, distance to the health facility, and literacy using both simple comparative measures and concentration indices. We also ran hierarchical random effects regression to confirm the presence or absence of inequities due to household wealth, distance, and literacy, while controlling for potential confounders.

**Results:**

Coverage of facility based delivery was high (89%), but suboptimal for at least four antenatal care visits (44%) and one postnatal care visit (53%). We detected inequities along the dimensions of household wealth, literacy and distance. Service coverage was higher among the least poor, those who were literate, and those living closer to a health facility. We detected a significant positive association between household wealth and all outcome variables, and a positive association between literacy and facility-based delivery. We detected a negative association between living farther away from the catchment facility and all outcome variables.

**Conclusion:**

Existing inequities in maternal health services in Burkina Faso are likely going to jeopardize the achievement of Universal Health Coverage. It is important that policy makers continue to strengthen and monitor the implementation of strategies that promote proportionate universalism and forge multi-sectoral approach in dealing with social determinants of inequities in maternal health services coverage.

## Background

There has been growing concern about inequities in health and health care at global, regional and country levels [1–5]. Evidence demonstrates that the poor and marginalized segments of society have the worst health status, as well as limited access to health enhancing interventions [3, 5–7]. Unfortunately, failure to address equity has been observed to be one of the most serious shortcomings of the Millennium Development Goals (MDGs), particularly of those pertaining to health such as reducing child mortality, improving maternal health, and combating HIV/AIDS, malaria and other diseases [8–11]. Many countries concentrated on reaching targets by acting to reduce mortality and morbidity at the national level, without necessarily addressing inherited inequities in the quest towards universal coverage [8, 12]. The MDG’s unfinished agenda has been picked up by the Sustainable Development Goals (SDGs), a set of goals meant to guide development efforts across sectors up to 2030 [11]. The adoption of Universal Health Coverage (UHC) as one of the targets for SDG 3 - healthy lives and well-being for all - is a very welcome development as equity is implicitly assumed to be included [13]. However, unless equity considerations related to access to and utilization of health care and health outcomes are explicitly accounted for in UHC policies, a risk remains that progress will be made only among the least poor segments of society, widening instead of narrowing the already existing equity gaps [14, 15]. Hence, the United Nations Committee on information and accountability for Women’s and Children’s Health suggests that indicators for reproductive, maternal and child health should be disaggregated using social stratifiers, such as wealth quintiles, gender, residence (urban/rural), and education, among others. This is considered essential to adequate monitoring of equitable progress towards achieving the SDG health indicator targets at all levels, from global to regional to country [2, 11].

Globally, there has been substantial progress in curbing maternal deaths such that between 1990 and 2015, maternal mortality declined by 44% [16]. However, maternal mortality still remains unacceptably high, especially in developing countries [16, 17]. The globally declining figures also mask large differences within world regions and country levels [16, 17]. A high burden of maternal mortality is increasingly concentrated in sub-Saharan Africa (SSA). Beyond inequities across countries and regions, important inequities within countries persist, whereby maternal mortality rates among the poor and the least educated women are twice as high as those among the least poor and the more educated women [18]. This situation follows from the fact that in SSA, the rates of skilled birth attendance - identified as the most important factor in reducing maternal deaths and an important element in reducing neonatal deaths [19, 20] - are also five times higher among the non-poor than among the poor [21]. In addition, there are also inequities in focused antenatal care, a service that has proved to provide opportunities for early detection of potential obstetric risks, and that through counseling and education, motivates women to seek skilled attendance at birth [19, 22, 23]. For example, use of at least four antenatal care visits differs by 25 percentage points between both the most and least educated, and the richest and poorest women [24]. Most maternal deaths occur within the first 24 h after birth [25] and 66% occur during the first week [26]. However, postnatal care services which could help to avert maternal and neonatal deaths reach even fewer women in SSA than in other world regions: less than half of women receive postnatal care within two days of childbirth, with rates being even lower among the poorer and less educated [27].

Burkina Faso is one of the countries in Sub-Saharan Africa that failed to achieve the target for MDG goal number 5 – reduction of maternal mortality by 75% between 1990 and 2015 [18]. However, Burkina Faso has made serious efforts towards ensuring equitable access to maternal care services. Several maternal health financing and delivery reforms were developed and implemented, among which are the abolition of user fees for antenatal care (ANC) services in 2002, subsidization of delivery costs for all women by 80% and by 100% for the poorest in 2007, and exemption of the poorest from payment of all user fees for all curative and preventive health services in 2009 [28, 29]. Despite some noticeable decline, the maternal mortality ratio still remains high at 371 per 100,000 live births [30]. Coverage of health services varies greatly across districts and between rural and urban areas [31, 32]. A few studies have investigated determinants of utilization and socio-economic inequities in using maternal health services. These studies, however, have focused on a few restricted geographical areas and focused only on specific services, failing to address the maternal care continuum and equity [33–35]. Conflicting evidence has emerged with regard to the role of household wealth in determining utilization of maternal health services. One study found that household wealth was negatively associated with utilization of ANC visits [33]. One explanation given for this negative relationship was that poor women might have benefited the most from the new financing policy (i.e. removal of user fees for ANC), while lower ANC utilization among the least poor could be attributed to low value attached to ANC coupled with unwillingness to endure the long waiting times which had resulted from increased utilization after abolition of user fees for ANC [33]. Another study found that household wealth was equity neutral in utilization of at least one ANC visit and facility-based delivery, but was positively and significantly associated with utilization of at least four ANC visits [34]. Inequities in utilization of ANC and facility-based delivery services were also found to be negatively associated with distance, animist religion and some ethnicities [33, 34]. As these studies were purely quantitative, they did not offer any explanation for the findings, calling for the application of further qualitative research [33, 34].

This study seeks to fill an existing gap in knowledge by exploring inequities and their determinants in utilization across the maternal care continuum in a large representative sample, including 24 districts in Burkina Faso. By doing so, the study aims at contributing a deeper understanding of whether and what inequities in access to and utilization of maternal care persist in the nation.

## Methods

### Study setting

Burkina Faso is a landlocked, francophone country in West Africa demarcated into 13 regions with 63 districts. In 2016, average life expectancy was estimated at 53.4 years for men and 57.6 years for women. In 2016, about 70% of the population were estimated to live in rural areas and only 36% to be literate [36]. Poverty is widespread, with about 41.1% of the population living below the national poverty line of US$1.90 a day [36]. The public health system in Burkina Faso is organized along three levels: primary level (named Centre de Santé et de Promotion Sociale - CSPS) in rural areas some urban areas, secondary level in district capitals and tertiary referral level in regional capitals and in Ouagadougou, the capital city. Total expenditure on health was at 5% of GDP in 2014 [37]. As noted earlier, Burkina Faso has made access to maternal and child health services one of its key policy objectives. It has done so by introducing a series of reforms, first to reduce (in 2007) and then to remove (in 2016) user fees for maternal care services [38–40]. While it is still early to evaluate the impact of the 2016 policy, evaluations of the 2007 user fee reduction policy indicate equity-neutral increases in health service utilization and decreases in out-of-pocket expenditure [41]. This is to say that the 2007 user fee reduction policy neither increased nor decreased existing gaps in service utilization between socio-economic strata, but kept them constant by improving access to care and financial protection across all socio-economic strata.

### Data sources

This study used data from a cross-sectional household survey conducted as part of the baseline assessment of the impact evaluation of a performance-based financing pilot intervention launched in Burkina Faso in 2014. Data was collected between October 2013 and March 2014 in 24 districts (38% of all districts in the country) on a mainly rural population (91.8%) – hence after the 2007 user fee reduction policy, but before the 2016 user fee removal policy. Sampling followed a three-stage clustering procedure. First, clusters were defined according to the catchment areas of 561 primary health facilities in the 24 districts. Second, one village was randomly selected from each cluster. Third, for each sampled village, teams of interviewers drafted a comprehensive list of all households with at least one woman who was either pregnant at the time of the visit or had completed a pregnancy in the prior 24 months. Subsequently, 15 households were to be randomly selected from the list for inclusion in the survey. The final sample included 7844 households, somewhat less than intended as it was not always possible to identify 15 eligible households per sampled village. For this study, we focus on the sub-sample of the 6655 mostly rural and poor women with a completed pregnancy in the prior 24 months residing in the sampled households [42]. However, each of the three outcome variables (i.e.: at least four ANC visits, facility-based delivery and at least one postnatal care) had a different sample size. This was due to the following reasons: i) some women included in the main sample had incomplete pregnancies such as abortions and miscarriages and hence did not attend any of the three maternal health services, and ii) some women attended only one service and not the other services along the continuum of maternal care, and iii) some women might have been missed due to either not completing giving responses to the questionnaire as the survey progressed or due to interviewer mistakes.

The survey questionnaire assessed households’ and women’s socio-demographic characteristics, and their use of essential maternal health care services during pregnancy (ANC, facility-based delivery, and postnatal care).

### Variables and their measurement

Table 1 provides an overview of all variables included in our analysis, their measurement, and their distribution in the sample.

**Table 1:** Variables considered in the analyses, their measurement, and distribution in the sample

Variable	Measurement	n (%)
Outcome Variables^a^		
At least 4 ANC^b^ visits (binary)	0 = Attended less than 4 ANC visits	3691 (55.9)
	1 = Attended at least 4 ANC visits	2910 (44.1)
Facility-based delivery (binary)	0 = Did not deliver at a facility	714 (10.9)
	1 = Delivered at a facility	5821 (89.1)
At least 1 postnatal care visit (PNC)^c^ within six weeks after birth (binary)	0 = Did not attend PNC1^d^	3067 (47.0)
	1 = Attended PNC1^d^	3459 (53.0)
Explanatory Variables		
Household Wealth Quintiles (categorical)	1 = Poorest	1315 (19.8)
	2	1267 (19.0)
	3	1291 (19.4)
	4	1352 (20.3)
	5-Least poor	1429 (21.5)
Literacy (binary)	0 = Illiterate	5168 (77.7)
	1 = Literate	1487 (22.3)
Distance of household to catchment facility	1= > 5 km	2651 (39.8)
	0 = ≤ 5 km	4003 (60.2)
Marital status (binary)	0 = Unmarried	188 (2.8)
	1 = Married	6445 (97.2)
Age (categorical)	1 = 15–20 years	1063 (16.0)
	2 = 21–29 years;	2920 (43.9)
	3 = 30–39 years	2079 (31.3)
	4 = 40+ years	585 (8.8)
Religion (categorical)	1 = Christian	1695 (25.5)
	2 = Muslim	4131 (62.1)
	3 = Other	828 (12.4)
Parity (categorical)	1 = 1 Pregnancy	1266 (19.4)
	2 = 2–3 Pregnancies	2322 (35.6)
	3 = ≥4 Pregnancies	2935 (45.0)
Number of household members (categorical)	1 = 1–3 Members	1274 (15.2)
	2 = 4–6 Members	3212 (48.3)
	3 = 7 Or more members	2168 (32.5)

^a^Outcome variables not adding up to 100% due to missing values as the survey proceeded from one section to the other

^b^ANC = Antenatal care; ^c^PNC = Postnatal care; ^d^PNC1 = at least one postnatal care visit

We defined our primary outcomes to capture coverage along the maternal care continuum, hence we included: a. having attended at least four antenatal care visits (ANC4+); b. having had a facility-based delivery, as a proxy measure for skilled attendance at delivery [43, 44]; and c. having attended at least one postnatal care visit (PNC1) within six weeks after birth.

Equity is defined as the absence of systematic disparities in health, its social determinants, and/or in health service utilization between more or less disadvantaged social groups [7, 45]. Inequities exist in the presence of disparities or determinants that are deemed avoidable, unfair and unjust [7]. In this study, equity refers to equal utilization of health services given equal need for such services [7], and when “need” is defined as the capacity to benefit from any service along the continuum of maternal health care. The literature recognizes multiple dimensions to equity in health service utilization, such as gender, wealth, education, place of residence/geographic region, ethnicity, age, migratory status, religion, occupation, indigenous status, or sexual orientation [12, 24, 46]. In this study, we investigated inequities in maternal health service coverage along three equity dimensions: i) household wealth; ii) woman’s education (measured in relation to literacy); and iii) distance to catchment primary health facility. Household wealth was selected, as overcoming socio-economic inequities in maternal health service coverage remains a top priority for the government and also a priority for the achievement of the SDGs [28, 29, 47]. In order to measure the household wealth, a wealth index using assets and living conditions was developed using the multiple correspondence analysis (MCA) [48]. The following variables were used to compute the household wealth index: housing (type of building materials, number of rooms, water and energy supply sources), assets (TV, radio, fridge etc.), house and fields owned, and animals. After calculation of wealth scores, households were split into quintiles from the poorest (Q1) to least poor (Q5).

Distance and literacy were chosen because they were identified as important barriers to access by earlier studies [22, 33, 34]. Distance was dichotomized so as to reflect the World Health Organization standard of having a primary health facility within a radius of 5 km as well as those living outside this recommended World Health Organization 5 km radius standard. Literacy was used instead of education: although this is recommended for equity analysis, in our sample, less than 1% of the respondents had formal education. Based on Andersen’s behavioral model [49], we included in our analysis a number of additional explanatory variables that were available in our data set as potential relevant confounders. These included: woman’s marital status, age, parity, and religion, as well as household size.

#### Analytical approach

Our analysis proceeded in steps. First, we looked at the differences in coverage for each of the three outcome variables by districts, and explanatory variables through descriptive bivariate statistics. The chi-square test was used to identify significant associations between the outcomes of interest and selected explanatory variables.

Second, to measure equity, we used simple comparative rates/measures of coverage for two groups [12, 50]. Simple comparative rates/measures draw on data from two subgroups and include differences and ratios to demonstrate absolute and relative inequalities, respectively [12, 46, 50, 51]. The absolute gap for socio-economic inequity was computed by subtracting the outcome of the first quintile from that of the fifth quintile (Q5-Q1) of the respective outcome variable. The ratio of socio-economic inequity was established by dividing the outcome of the fifth quintile to that of the first quintile (Q5/Q1), respectively. Because distance and literacy were binary variables, we computed inequity gap and ratio in the same way as with the continuous variable. It is important to note that absolute measures provide an idea of the actual gap that exists between groups and thus the required effort to close them while relative measures provide an insight into the degree of unfairness between groups [52]. To correct for the weaknesses of the simple comparative rates, especially for socio-economic position (since they only take into account the two extreme groups, leaving out other groups in the middle [53]), we used concentration indices, which are estimated using concentration curves, to draw on data from more than two subgroups [12, 46, 51, 54]. Concentration curves provide a graphical display of the share of health or health services accounted for by cumulative proportions of individuals in a population ranked from poorest to richest at a given point in time [51, 55]. A concentration curve that lies below the line of equality (45 degrees) signifies presence of inequality favouring the rich, while a curve that lies above the equality line signifies presence of inequality favouring the poor. When it overlaps with the diagonal line (the line of equality), this implies there are no inequalities [51, 55]. Concentration indices quantify the degree of socioeconomic-related inequality in a given health or health service variable [51, 55], defined as twice the area between the concentration curve and the diagonal (line of equality) ranging between − 1 and 1. The index takes a negative value when the curve lies above the line of equality, indicating disproportionate concentration of the health or health service variable among the poor and a positive value when it lies above the line of equality, indicating disproportionate concentration of the health or health service variable among the rich and takes the value of zero when there is equality [56].

Third, we ran three separate regressions (one per outcome variable) to confirm the presence or absence of inequities due to household wealth, distance, and literacy, while controlling for all potential confounders. As such, we performed a regression analysis using a hierarchical model to allow for clustering at the district level, attempting to capture the variance in the outcome variables across districts captured by the descriptive analysis. We operationalized our random effects models using Stata version 14 (Stata Corporation, Texas, USA), defining women as first level and district as second level. Our estimated model is of the form:

1 $$ {y}_{ij}={\beta}_{0j}+{\beta}_j{x}_{ij}+{u}_j+{\varepsilon}_{ij} $$

where of each observation ‘i’, Y is one of the 3 outcome variables ‘j’ [j = 1(ANC4+, 2(facility-based delivery), 3 (PNC1)] and X is the explanatory variables, *β*_0*j*_is the intercept of the respective model for outcome variable ‘j’, *u*_*j*_ is the district-specific effects, and *ε*_*ij*_ is the error term. As is the case with hierarchical models, our assumption is that each of the levels (districts) has a different (i.e. district-specific) effect *u*_*j*_ on the outcome variables *y*_*ij*_, which are independent of the explanatory variables *x*_*ij*_.

## Results

Table 1 shows the descriptive statistics for all variables included in the analyses. Facility-based delivery had the highest coverage (89%), followed by use of PNC1 (53%). ANC4+ had the least coverage (44.1%).

Table 2 shows the results of coverage measured by our outcome variables in relation to districts. The results show that there was great variation in the coverage of the three service types across the districts. This ranged from a low of 21% in Yako in Nord region to a high of 66% in Tenkodogo in Centre-Est region for ANC4+; from a low of 64% in Gaoua in Sud-Ouest region to a high of 100% in Ziniare in Plateau region for facility-based delivery; and from a low of 34% in Gaoua in Sud-Ouest region to a high of 75% in Nanoro in Centre-Ouest region for PNC1.

**Table 2:** Coverage of ANC4+, facility based delivery and PNC1 by district

	ANC4+ visits		Facility based delivery		PNC1	
	(N)	%	(N)	%	(N)	%
Total	6601	44.1	6535	89.1	6526	53.0
Region/District						
Boucle du Mouhoun						
Boromo	144	48.6	143	88.1	143	62.2
Nouna	542	50.4	540	88.5	540	51.5
Solenzo	406	46.3	402	85.1	401	36.9
Toma	137	45.3	135	88.1	135	67.4
Centre-Sud						
Manga	110	45.5	110	96.4	110	41.8
Centre-Est						
Ouargaye	303	56.1	300	96.0	296	41.2
Tenkodogo	268	66.0	268	98.9	268	43.7
Zabré	38	63.2	38	97.4	38	44.7
Centre-Nord						
Barsalogho	59	33.9	59	91.5	59	59.3
Kaya	643	42.3	634	85.3	634	67.0
Kongoussi	425	39.4	420	94.1	419	72.1
Plateau Central						
Ziniaré	252	60.3	250	100	250	41.2
Boussé	163	38.0	162	96.3	162	39.5
Centre-Ouest						
Koudougou	777	48.3	769	89.3	769	70.7
Nanoro	77	39.0	76	89.5	76	75.0
Réo	194	33.5	194	85.6	194	47.4
Sapouy	249	41.4	245	84.9	245	52.7
Nord						
Gourcy	377	39.8	377	95.5	377	43.8
Ouahigouya	748	33.6	734	85.3	733	42.4
Yako	204	20.6	203	94.1	203	37.0
Sud-Ouest						
Batié	116	46.6	110	67.3	108	46.3
Dano	55	41.8	55	87.3	55	67.3
Diébougou	247	41.7	244	79.1	244	57.0
Gaoua	67	38.8	67	64.2	67	34.3
*P-value (District)*	*0.000*		*0.000*		*0.000*	
Chi2 test						

Table 3 presents results of service coverage measured by our three outcome variables in relation to our main equity measures and all additional explanatory variables. Bivariate analysis detected few statistically significant differences among subgroups for ANC4+ coverage, but detected many statistically significant differences among subgroups in respect to coverage for facility-based delivery and PNC1. Coverage of ANC4+ was higher among women from least poor households (*p* ≤ 0.1), women who lived near a primary health facility (*p* ≤ 0.001) and women who were literate (p ≤ 0.1). Facility-based delivery and PNC1 were higher among women from the least poor households (p ≤ 0.001), women living near a primary health facility (p ≤ 0.001) and literate women (p ≤ 0.001).

**Table 3:** Coverage of ANC4+, facility-based delivery and PNC1 by population subgroups

	ANC4+		Facility-based delivery		PNC1	
	N	%	N	%	N	%
Total	6601	44.1	6535	89.1	6526	53.0
Household Wealth Quintiles						
Poorest	1307	41.6	1298	84.3	1295	48.7
2	1250	43.6	1239	88.9	1239	48.1
3	1278	43.1	1265	88.8	1263	54.9
4	1346	44.5	1335	90.2	1335	53.9
Least poor	1421	47.1	1398	92.9	1394	58.9
*P-value*	*0.059*		*0.000*		*0.000*	
Distance of household to catchment facility						
≤ 5 kms	3976	47.2	3937	93.0	3932	54.5
> 5 kms	2626	39.2	2598	83.1	2594	50.8
*P-Value*	0.000		0.000		0.000	
Literacy						
illiterate	5127	43.4	5078	88.2	5070	51.7
literate	1475	46.2	1457	92	1456	57.4
*P-value*	0.062		*0.000*		*0.000*	
Marital Status						
Married	6393	44.3	6331	89.3	6322	53.0
Not Married	188	37.2	184	80.4	184	54.9
*P-value*	0.055		*0.439*		0.610	
Age						
15–20	1053	43.5	1043	90.9	1043	57.9
21–29	2896	44.3	2864	89.8	2861	53.1
30–39	2066	44.1	2050	88.3	2047	51.8
40+	580	44.1	572	85.0	571	48.0
*P-value*	0.979		*0.001*		0.001	
Parity						
1	1255	46.8	1245	92.7	1243	55.8
2--3	2305	43.4	2284	89.1	2284	52.5
≥ 4	2916	43.2	2894	87.4	2889	52.3
*P-value*	0.079		*0.000*		0.050	
Religion						
Christian	1686	46.26	1671	90.0	1668	57.1
Moslem	4098	43.19	4057	90.6	4052	52.1
Other	818	43.77	807	79.4	806	49.4
*P-value*	0.100		*0.000*		*0.000*	
Number of household members						
1—3 members	1258	44.99	1238	90.2	1237	55.1
4—6 members	3188	43.44	3160	88.9	3156	54.1
≥ 7 members	2156	44.39	2137	88.7	2133	50.2
*P-value*	0.598		*0390*		0.006	
Chi2 test						

Table 4 presents the results of inequities related to maternal health service coverage of the three outcome variables measured using simple comparative rates/measures of coverage. With regard to socio-economic position, the absolute inequity gap in coverage between the least poor (Q5) and the poorest (Q1) was widest in PNC1 (10.3 percentage points), followed by facility-based delivery (8.6 percentage points) and lastly ANC4+ (5.5 percentage points). Coverage was higher among literate women with an absolute gap of 2.7 percentage points for ANC4+, 3.8 percentage points for facility-based delivery, and 5.7 percentage points for PNC1. Coverage was also higher among women living close to health facilities with an absolute gap of 8 percentage points for ANC4+, 9.9 percentage points for facility-based delivery and 3.7 percentage points for PNC1. The rate ratios in respect to coverage for the three services and the three equity dimensions are also presented in Table 4. For instance, the results show that coverage of ANC4+ and facility based delivery were 1.1 times higher among the least poor than among the poorest, and 1.2 times higher among the least poor than among the poorest for PNC1. Results further show that coverage of ANC4+ and PNC1 were 1.1 times higher among the literate than among the illiterate; and for facility-based delivery, there were no differences between the literate and the illiterate. With regard to distance, coverage of facility-based delivery and PNC1 were 1.1 times higher among those living near a health facility compared to those living far away, while coverage of ANC4+ was 1.5 times higher among those living near a health facility compared to those living far away.

**Table 4:** Comparison of Inequities in coverage of ANC4+, facility-based delivery and PNC1 along dimensions of Household wealth, Literacy and Distance

Outcome variable	Mean (%)	Health Equity Dimension					
		Household wealth		Literacy		Distance^a^	
		Absolute Difference (percentage point)	Ratio	Absolute Difference (percentage point)	Ratio	Absolute Difference (percentage point)	Ratio
		Least poor-Poorest	Least poor/ Poorest	Literate-Illiterate	Literate/ Illiterate	Near-Far	Near/Far
ANC4 + visits^b^	44.1	5.5	1.1	2.7	1.1	8.0	1.5
Facility-based delivery	89.1	8.6	1.1	3.8	1.0	9.9	1.1
PNC1^c^	53.0	10.3	1.2	5.7	1.1	3.7	1.1

^a^Near = ≤5 km to health facility and Far= > 5 km to health facility, ^b^ANC4 + =at least four antenatal care visits, ^c^PNC1 = at least one postnatal care visit

Basic source: Table 3

The concentration indices also confirmed that inequities in coverage between the least poor and the poorest were largest for PNC1 with a value of 0.0415 (*p* < 0.001), followed by facility-based delivery with a value of 0.0181 (*p* < 0.01). Coverage of ANC4+ displayed the least inequity with a concentration index of 0.0239 (*p* > 0.05). All indices were positive and statistically significant except for ANC4+ visits, indicating the existence of inequalities along the maternal care continuum in favour of the least poor for PNC1 and facility-based delivery.

Table 5 presents the hierarchical regression analysis results for the three outcome variables. Model 1 confirms the presence of inequities in coverage of ANC4+ linked to household wealth, distance, marital status, parity and religion (these were variables with statistically significant results). Women from the least poor households compared to women from poorest households and women who were married compared to women who were not married were significantly more likely to use ANC4+ visits. Women living further away from a primary health facility compared to women living near a primary health facility, women with 4 or more pregnancies compared to women with one pregnancy and women of Muslim religion compared to women of Christian religion were significantly less likely to use ANC4+ visits.

**Table 5:** Determinants of coverage of ANC4+, facility-based delivery and PNC1

Variable	Model 1		Model 2		Model 3	
	ANC4+		Facility based delivery		PNC1	
	Coefficient	Std Error	Coefficient	Std Error	Coefficient	Std Error
Household wealth Quintiles						
Poorest –Reference						
2	0.0510	(0.0838)	0.2836*	(0.1274)	0.0628	(0.0846)
3	0.0678	(0.0845)	0.2789*	(0.1287)	0.3710***	(0.0857)
4	0.1292	(0.0850)	0.4542***	(0.1347)	0.3264***	(0.0863)
Least poor	0.2108*	(0.0859)	0.7819***	(0.1455)	0.5312***	(0.0882)
Literacy						
Illiterate –Reference						
literate	0.1199	(0.0638)	0.2665**	(0.1140)	0.0905	(0.065)
Distance to catchment facility						
≤ 5 km –Reference						
> 5 km	−0.3382***	(0.0557)	−1.0295***	(0.0900)	−0.1598***	(0.0567)
Marital status						
Married	0.3250*	(0.1620)	0.3418	(0.2183)	0.1591	(0.0656)
Age (years)						
15–20-Reference						
21–29	0.1513	(0.0920)	0.1008	(0.1589)	−0.2340*	(0.0941)
30–39	0.1768	(0.1098)	0.0800	(0.1842)	−0.3084**	(0.1121)
40+	0.1812	(0.1360)	−0.2187	(0.2164)	−0.4470***	(0.1389)
Parity						
Parity = 1-Reference						
Parity = 2–3	−0.1232	(0.1015)	−0.6130***	(0.1808)	−0.0419	(0.1043)
Parity = ≥4	−0.1649*	(0.1143)	−0.7174***	(0.2015)	−0.0446	(0.1173)
Religion						
Christian-Reference						
Muslim	−0.1322*	(0.0658)	0.0063	(0.1133)	−0.0586	(0.0679)
Other	−0.0031	(0.0971)	−0.3395**	(0.1406)	−0.2877**	(0.1001)
Household Members						
1–3-Reference						
4–6 members	−0.0546	(0.0957)	0.1808	(0.1568)	0.0418	(0.0981)
≥ 7 members	−0.0390	(0.1079)	0.0543	(0.1764)	−0.1532	(0.1104)
_cons	−0.4503*	(0.1964)	2.6269***	(0.3143)	0.00727	(0.2094)
/lnsig2u	−1.9015	(0.3412)	−0.3680	(0.3754)	−1.3576	(0.3250)
sigma_u	0.3865	(0.0659)	0.8319	(0.1562)	0.5072	(0.0824)
rho	0.0434	(0.0141)	0.1738	(0.0539)	0.0725	(0.0219)
LR ratio test of rho, chibar2(01)	150.65***		140.45***		327.62***	
Wald chi2(19)	64.74***		223.60***		103.43***	

*p < 0.05; **p < 0.01; ***p < 0.001

Standard errors in parenthesis

Likewise, Model 2 confirms the presence of inequities in coverage of facility-based delivery linked to household wealth, distance, literacy, parity and religion (these were variables with statistically significant results). Women from the least poor households compared to women from poorest households and literate women compared to illiterate women were significantly more likely to use facility-based delivery services. High parity (4 or more pregnancies) compared to low parity (one pregnancy), women from other religions compared to Christianity and women living farther away from the primary health facility compared to women living near a primary health facility were significantly less likely to use facility-based delivery services.

Furthermore, Model 3 confirms the presence of inequities in coverage of PNC1 linked to household wealth, distance, age and religion (these were variables with statistically significant results). Women from the least poor households were significantly more likely to use PNC1 than women from the poorest households. Women living farther away from a primary health facility compared to women living near a primary health facility, older women (21–29, 30–39, 40–49 years) compared to young women (15–20 years) and women belonging to other religions compared to Christianity were significantly less likely to attend PNC1.

With rho values of 0.0434 (CI 0.0227–0.0814), 0.1738 (CI 0.0916–0.3051) and 0.0725 (CI 0.0397–0.1288) at 95% CI for ANC4+ visits, facility-based delivery and PNC1, respectively, our models confirmed a considerable portion of the observed variation in coverage across districts was attributable to district-level variance. Due to the lack of information on district-level characteristics, we could not attempt to explain this variance.

## Discussion

This study investigated coverage of key maternal health services in respect to inequity and its determinants. The strength of this study lies in the comprehensive equity analysis along the continuum of maternal care services, drawing on a large sample of 6655 mostly rural, poor women from across 24 districts. This is unique, within and beyond the context of Burkina Faso, given that most existing studies focused more narrowly on just one or two maternal care indicators [33, 34, 57–59] and/or were restricted to specific/single locations [33–35, 38, 39, 60].

Results show that maternal health service coverage in Burkina Faso especially for facility-based delivery was high (89.1%), and is one of the few countries in SSA (others being Malawi 89%, Benin 87%, Gabon 90%, and Congo 92% [61]) to have achieved this high rate. However, coverage remains suboptimal, especially for those maternal health services that require multiple interactions with health care workers, such as ANC4+ (44.1%). Our findings on ANC4+ coverage of below 50% are consistent with the 2010 Demographic and Health Survey results on Burkina Faso and in sub-Saharan Africa where only 34 and 50% of women, respectively attended four or more antenatal visits [62]. PNC1 coverage results from this study are lower than those of the national estimates of 72% in 2010 [62]. However, this low coverage was consistent with the sub-Saharan African average of 50% [63].

In addition, our results indicate the persistence of large inequities in coverage across districts, confirming the inequitable patterns in coverage reported in the annual statistical reports compiled using data from the health management information system [31]. Coverage inequities across districts are probably attributable to district-specific characteristics, such as factors related to population density and quality of care on offer, which should be unpacked by future research. What is clear from our analysis, however, is that policies aimed at reducing financial barriers to access, such as user fee removal for ANC in 2002 and subsidies for facility-based delivery in 2007 [64], have not been sufficient to ensure optimal coverage rates nor to compensate for pre-existing inequities in coverage across districts. Future research will have to explore whether further steps taken by the government after 2013, such as the introduction of performance-based financing (with systematic targeting and exemption mechanisms for the poor) in 12 districts in 2014 and the full removal of user fees for all care delivered to pregnant and lactating women in 2016, will lead to expected results and be sufficient to close the inequities across districts we observed in our study.

Looking more specifically at the primary objective of our study, i.e. exploring inequities in coverage due to household wealth, distance, and literacy, our findings indicate the persistence of inequities across the entire maternal care continuum for ANC4+, facility-based delivery and PNC1 due to household wealth and distance. Inequities due to literacy were only prevalent in facility-based delivery. Women from the least poor households compared to women from poorest households and women living close to a primary health facility compared to women living farther away from a primary health facility had higher service coverage across all maternal care services. In addition, literate women compared to illiterate women had higher coverage of facility based delivery. Socio-economic inequities have been reported both by earlier studies in Burkina Faso [34, 65] and elsewhere in other developing countries such as Bangladesh [14, 58, 66], Afghanistan [52], India [60], Malawi [67], Ghana [68], Ethiopia [69] and Namibia [53, 70]. In fact, household wealth has been found to be the most crucial factor in determining who receives maternal health services [22, 28, 71]. Interestingly, however, the magnitude of inequalities due to socio-economic position (household wealth) detected in our study is smaller than the one detected in earlier studies and elsewhere in sub-Saharan Africa [65]. For example the absolute gap in ANC4+ coverage between the least poor and the poorest in Burkina Faso Ghana, Cameroon, Senegal, Côte D’Ivoire and Gabon ranged from 23 to 46- percentage points [65], compared to the 5.5 percentage points detected in our study. Similarly, the absolute gap in facility-based delivery coverage between the least poor and the poorest in Burkina Faso Ghana, Cameroon, Senegal, Côte D’Ivoire and Gabon ranged from 20 to 78 percentage points [65], compared to the 8.6 percentage points detected in our study. This discrepancy between prior findings and our study is probably largely due to the almost complete absence of wealthier urban women from our sample. It could be argued that countries which had similar policies to Burkina Faso of subsidizing deliveries, might have reduced socio-economic inequity gaps even though this led to equity-neutral increases in Burkina Faso as noted earlier. Evidence indicates, however, that Ghana which implemented a similar policy at the same time as Burkina Faso, experienced greater inequity in coverage of facility-based deliveries between socio-economic groups [72].

One can therefore safely assume that the differences in magnitude of inequities between earlier studies and our study mainly arise from our sample which, is largely rural and on average is poorer than samples including more urban women, such as those of the Demographic and Health Surveys (DHS). Nevertheless, albeit smaller in terms of absolute magnitude, the inequities in socio-economic position detected by our analysis are worthy of attention.

Low coverage rates among the poorest women suggest a certain inability on the part of the policies in place to compensate for the disadvantage linked to poverty. This consideration is worrisome given that health outcomes are often at their worst among the poorest population that the literature identified as also having the greatest need [6, 7, 73, 74]. It follows that health policies should be specifically targeted to compensate for existing inequities due to socio-economic position by actively encouraging service use among the poorest. Evidence shows that countries that have made rapid progress in maternal health services coverage were those that effectively reached the poorest [15]. Prior research, however, has already indicated that due to an implementation gap in the application of the complete fee exemption for the poorest, the 2007 subsidy for deliveries and obstetric care policy has resulted in an equity-neutral improvement in service use and out-of-pocket spending, but has failed to close existing equity gaps as originally envisioned [75, 76]. It remains to be seen whether the targeting component (including an exemption mechanism for the poorest) embedded in the abovementioned performance-based financing intervention will be faithfully implemented up to the end of the pilot and result in a reduction in inequities due to socio-economic position [77, 78].

Our findings suggesting that living farther away from a health facility represents an important source of inequity in access for all maternal care services are consistent with earlier studies conducted in Burkina Faso [35, 79] and in Ghana, Nigeria, Sierra Leone [80, 81] and Ethiopia [69]. Evidence shows that poor women face several additional barriers in the utilization of maternal health services due to the long distance to health facilities, poor road conditions, absence of a well-organized transport system, and indirect or direct costs associated with transport [22, 82–84].

Similarly, the inequities due to literacy detected in our study are consistent with existing evidence [22, 33–35, 65, 71, 85]. Literacy, like education, increases the knowledge of the health benefits of preventive care and awareness of health services, improves the ability of individuals to attain health by influencing their life style, and increases the use of health services through improved knowledge, attitude and practice [59, 71, 86]. Unfortunately, the less educated or illiterate require more education to appreciate the value of health services [43]. Coupled with other challenges such as poverty, maternal health services coverage among the illiterate is generally low [87].

Findings from our regression analysis indicated the role played by an additional set of demand-side factors in shaping service coverage along the maternal care continuum. In line with prior studies [58, 59, 68, 88–93], for instance, we found that parity (having 4 or more pregnancies compared to one pregnancy), marital status (being unmarried compared to being married), and religion (being Muslim as compared to Christian) decreased the likelihood of completing ANC4+ visits. In addition, other traditional religions affected the extent to which facility-based delivery and postnatal care services are being used. Furthermore, parity (having 4 or more pregnancies compared to one pregnancy and age (being older (21–29, 30–39, 40–49 years) compared to being younger (15–20 years) affected the extent to which facility based delivery and PNC1 visit are being used, respectively. These findings are also consistent with some studies conducted in Burkina Faso [33, 35] and in other developing countries such as India and Ghana [92, 94] where certain religious groups make less use of certain maternal health services; Ethiopia and Pakistan [69, 95] where high number of pregnancies are negatively related to use of certain maternal health services, rural China [96], where higher age is negatively related to use of maternal health services; and rural Vietnam [97], where being unmarried is negatively related to use of certain maternal health services.

Furthermore, our multivariate analysis did not identify literacy as a significant determinant of ANC4+ and PNC1, albeit originally significant in bivariate relationships. This suggests the existence of a probable positive correlation between literacy and household wealth, as noted in prior studies [98]. Still, given that the literature also suggests an independent role of literacy on health outcomes [98]; and given that literacy remained a significant determinant of facility-based delivery, we reiterate the importance for policies to address both socio-economic and literacy barriers.

In addition, our study used the same set of explanatory variables to estimate the three outcome variable models. Important differences in association between maternal health services and these explanatory variables have been observed. The observed associations differed across the three regression models, indicating that decisions to seek services along the continuum of maternal care are shaped by different factors. Thus addressing such determinants of inequities requires a holistic approach along the continuum of maternal health care. In addition, some of these issues are beyond the health sector mandate. These social determinants of health need to be addressed through a multi-sectoral approach in line with the recommendations of the Commission on Social Determinants on Health [5].

To further reduce socio-economic inequities, policy makers could strengthen proportionate universalism -the resourcing and delivering of universal services at a scale and intensity proportionate to the degree of need [99, 100]. In so doing, services would be universally available, not only for the most disadvantaged, and are able to respond to the level of presenting need [99]. In addition, policymakers could consider introducing new financing and delivery reforms targeting the poorest women, such as conditional cash transfers and vouchers, which have proved to be successful in reducing socio-economic inequities in the coverage of maternal health services in other countries such as Bangladesh, Kenya, Pakistan, India and Cambodia [44, 66, 95, 101, 102]. Government could further consider strengthening community based outreach services, especially for services like antenatal and postnatal care [24, 103]; and bottom up approaches to identification of health problems and implementation strategies that deal with health problems affecting the poor through community engagement and collaborative research [104–106]; and strengthen health literacy in order to deal with other additional determinants of inequities. This in turn could improve women’s knowledge on preventive care benefits, awareness of health service use, lifestyle changes, personal attitudes and practices [86].

In this study, there are a few limitations that must be noted. First, we need to acknowledge that our findings may be affected by recall bias [107, 108], since women were asked to report on service use that could have taken place more than two years prior to the survey date. However, it is unlikely that this had much effect as the interviewers were well trained on how to describe the time period in which this study fell and the interviewers were also looking at the ANC booklets to verify the reported information. Second, the use of facility-based delivery as a proxy of skilled birth attendance could raise some concerns that the two are not the same. However, it has been found that in developing countries professional/skilled delivery care is nearly synonymous with facility-based care in most countries, with a few exceptions such as Haiti, Indonesia and Madagascar, where home delivery with a professional is relatively common [43]. Third, the measure of socio-economic position may be called into question - the household asset wealth index not having been very sensitive since this is a rural sample and people are all poor, unlike in a situation whereby the proportion of the least poor in the sample is also large. Evidence shows that in most low-and middle-income countries, households in the wealthiest quintile are often associated with urban areas, such that wealth inequalities are closely associated with urban/rural differences [109]. However, since we detected socio-economic differences in our study, this shows that our measurement was probably sensitive enough. Fourth, there were some, few cases in which residents of Burkina Faso in border districts with Côte D’Ivoire and Ghana obtained maternal health care services from these countries and were just coded by the name of the country without mentioning the type of service used. We treated such cases as seeking maternal health care from the informal sector hence counted as none service use.

## Conclusions

Coverage of facility based delivery in Burkina Faso is very high and comparable to only a few countries in sub-Saharan Africa. However, coverage remains suboptimal for ANC4+ and PNC1 like in many other sub-Saharan African countries. It is also clear that there is inequality favouring the least poor regarding maternal health service coverage. Thus, in order to achieve UHC, there is a need to review the existing pro-poor strategies and strengthen the development, implementation and monitoring of additional strategies such as those that promote proportionate universalism and bottom up and community engagement and collaborative research. In addition, intensify multi-sectoral approaches and health literacy campaigns in order to overcome other social determinants of inequities in coverage of maternal health services.

## Abbreviations

ANC: Antenatal care
CSPS: Centre de Santé et de Promotion Sociale – CSPS
MDG: Millennium Development Goals
SDG: Sustainable Development Goals
SSA: Sub-Saharan Africa
UHC: Universal Health Coverage
ANC4+: At least 4 antenatal care visits.
PNC1: At least one postnatal care visit
MCA: Multiple Correspondence Analysis
DHS: Demographic Health Surveys
